# Explaining psychosocial care among unaccompanied minor refugees: a realist review

**DOI:** 10.1007/s00787-021-01762-1

**Published:** 2021-03-29

**Authors:** Hanna-Sophie Ulrich, Emma Kohler, Jacob Spallek, Matthias Richter, Daniel Clauß, Martin Mlinarić

**Affiliations:** 1 Institute of Medical Sociology, Medical Faculty, Martin Luther University Halle-Wittenberg, Magdeburger Str. 8, 06112 Halle, Saale Germany; 2 Department of Public Health, Institute for Health, Brandenburg University for Technology Cottbus-Senftenberg, Senftenberg, Germany; 3 Department of Surgical and Conservative Pediatrics and Youth Medicine, University Hospital and Polyclinic for Pediatrics, University Hospital Halle, Saale, Germany

**Keywords:** Psychosocial care, Migration, Unaccompanied minor refugees, Realist review, mental health

## Abstract

Research on the psychosocial care (PSC) of unaccompanied minor refugees (UMRs) has mainly taken a socioepidemiological approach and has focused on the perspectives of experts in the field. In contrast, the knowledge concerning the differing context factors and the underlying mechanisms of current PSC which could inform policy recommendations is scant. The study aims at unravelling the contexts, mechanisms and outcomes of PSC for UMRs. For a realist review (RR), scientific evidence and gray literature were synthesised consistent with the RAMESES publication standards for realist synthesis. Based on an iterative keyword search in electronic databases (e.g., PubMed) and screening, 34 works from 2005 to 2019 were included in a realist synthesis. Theory-informed context–mechanism–outcome configurations (CMOs) were extracted, to explain underlying processes and mechanisms. Characterised by their interrelatedness, the dominant CMOs included the UMRs’ intersections of transitions (e.g., adolescence and migration), their needs for culture-, and gender-sensitive PSC, and the undersupply of PSC. These contexts and outcomes are mediated by pre-, peri- and post-migratory stressors as well as care structures and are moreover influenced by overarching discourses and concepts. They comprise adverse and beneficial mechanisms in the PSC of UMRs. The existing literature grasps the PSC of UMRs by different disciplines and approaches but does not offer a comprehensive overview on micro–macro intersections and included discourses. The inclusion of lay perspectives and an intersectional approach could inform health service research. The reflection of UMR-related categorical constructs of resilience and vulnerability, discourses of othering, as well as restrictive health policies may guide policy recommendations.

**Supplementary Information:**

The online version contains supplementary material available at 10.1007/s00787-021-01762-1.

## Introduction

In the context of the development of posttraumatic stress disorder (PTSD), depression, anxiety and several somatic (co)morbidities, unaccompanied minor refugees (UMRs) belong to a so-called “vulnerable” [1] high-risk group for these psychologically and psychiatrically relevant disorders [2–4]. Their experiences before and during migration and the fact that they enter receiving countries without family members can cumulate with other determinants of health, such as age (≥ ≤ 16 years), gender [5] and residential permit status. Protective factors and UMR supporting actions gain importance here and can be summarized as psychosocial care (PSC). European states, such as Belgium, the United Kingdom, Sweden and France, have reported a high increase in applications for asylum by UMRs [6]. In the years 2015 and 2016, Germany received the highest number of UMR asylum seekers (90% of them male) and according to the youth welfare offices the number of UMRs increased from 3000 in 2010 to approximately 45.000 in 2016 [7]. In 2018, the number of UMRs entering Germany declined to 12,201 [8].The current humanitarian crisis on the Greek islands and the statements of some European countries, such as Germany and France, suggest that the number of UMRs in 2020 and 2021 will increase again [9]. This review examines the PSC of UMRs.

Within the European context, PSC structures and the related policies differ greatly, making it difficult currently to standardise them. The supranational and national legal levels are, thus, legally binding, guiding and enforceable and can therefore be compared with each other, as for the German example (Table 1).

**Table 1:** Policies regarding PSC for UMR on European and German Level

European legal level	German legal level
Charter of Fundamental Rights of the European Union, Art. 24 & UN Convention of the Rights of the Child	Youth Welfare Act (SGB VIII) → Child and Youth Services Further Development Act 2005 (SGB VIII) Guardianship regulation (BGB §1771)
Legal guarantee of family reunification (EU 604/2013, Art 6, para 3a)	Exclusion of persons eligible for subsidiary protection from family reunion (Residence Act §104, 13)

The example shows the difference between EU-wide regulations and possible exclusions of PSC-relevant conditions within actual praxis. Given the 1989 UN Convention ofthe Rights of the Child, children are granted the right to adequate protection and humanitarian aid. The Charter of Fundamental Rights of the EU states that children are entitled to the protection and care necessary for their well-being, and all actions related to children must follow the best interests of the child. The German regulations of the youth welfare act include that every young person has the right to be supported in his or her development and be educated to become a self-reliant and community-oriented personality (§1 Abs. 1 SGB VIII). The Act was clarified in 2005 with the Child and Youth Services Further Development Act, which entitles UMRs for guardianship. The ambivalence of EU-wide and national regulations is evident in Germany, where residence status determines the possibility of a family reunion. The Europe-wide regulation specifies, in assessing the best interests of the child, Member States shall cooperate closely, taking due account in particular of the following factors: possibilities for family reunification and legal guarantee of family reunification[10]. In juxtaposition to this guarantee, the Residence Act of 2016, as part of asylum law in Germany, excludes persons eligible for subsidiary protection (to which UMRs can belong) from a family reunion [11].

PSC can be defined as services that give support within the psychological and social field and contribute to preventing the hardening of mental disorders and psychological distress. Thus, psychosocial care services include psychological counselling, therapy and psychiatric short- and long-term interventions. In this context, mental health is understood as “a state of well-being in which the individual realises his or her own abilities, can cope with the normal stresses of life, can work productively, and is able to contribute to his or her community” and implies psychological well-being and the absence of psychiatric disorders [12]. Alongside psychological and psychiatric support, PSC promotes integration and social participation because it aims to improve and stabilise the physical and mental state of health. The provision of support in bureaucratic procedures can serve as an example here [13].

The studies conducted in the German context show that PSC of UMRs is provisionally administered by emergency and refugee assistance [14] in so-called “clearing houses” [2] and legally administered by youth and social services. Furthermore, health service research has identified several cultural, legal, and structural barriers in healthcare access and utilisation for (adult) refugees [15–17]. UMRs have been scarcely studied in this context [5]. Most of the existing evidence comes from the psychological and epidemiological fields of health service research and underlines an increased need for PSC among UMRs [18, 19]. The legal, social and cultural mechanisms that form care structures have not yet been presented in a comprehensive way. The literature showed that psychosocial processes and mental health care are of great importance for UMRs and cannot be investigated separately.

Regarding the UMRs’ access to PSC, questions on how this access is contextualised (e.g., legally) and how it functions in practice as well as which barriers exist, has not yet been asked in this scope. We know that due to local contexts, the UMRs’ different personalities and diverging structural conditions, the UMRs’ PSC access and utilisation is not extensively comparable. Therefore, the existing literature offers diverging selective analyses (e.g., concentrating on female UMRs and their specific health care needs), theoretical discussions and region-specific empirical findings. An overview on how PSC is generally structured and which barriers and beneficial aspects exist is so far missing. The following realist review (RR) examines contexts, mechanisms, and outcomes of PSC for UMRs and will enrich the existing body of literature by trying to analyse realistic processes. It thus synthesises the existing findings on UMRs PSC.

## Methods

The RR methodology was applied to reveal the inner workings of PSC and its strategy to improve the mental, social and physical well-being for UMRs. Following the basic pillars of critical realism, by working with methodologically ideal–typical context–mechanism–outcome configurations (CMOs), a RR aims to unravel causal mechanisms [20, 21].

The RR approach [20] offers a methodological tool to study complex PSC contexts, mechanisms and outcomes for UMRs in the European context. Realist-informed CMOs can be considered as processes with contexts and outcomes constantly altering and reformulating social reality. A definition of CMOs within the RR method and the ways to elaborate them is described and illustrated in De Souza 2013 [22]. As a synthesis of existing evidence [20], the RR allows the theoretical inclusion of professionalised and daily discourses and concepts regarding UMRs. Moreover, through the use of comprehensive CMOs and with joining the findings together coherently, it aims at summarising the key findings. These CMOs will be used to formulate critical (non-discriminatory) and practical policy recommendations.

In this study, the realist approach investigates the *mechanisms* (M) of *how* and *why* PSC programs and contexts (C) may produce multiple *outcomes* (O) for UMRs. The term context in this regard denotes conditions, structures and cultures that may cause adverse or beneficial mechanisms. For instance, under asylum and youth care policies, the access of UMRs to a PSC may be hindered or fostered depending on the age and citizenship of the UMR [23]. Accomplished transformations that move from context to outcome or that are circular can then affect agency, relations and practices [22] because different factors are associated or cease to work. As an example, limited access to care services, such as regular psychiatric care, can lead to UMRs having to go to other care facilities. As ideal–typical constructs, realist CMO configurations are presented in a mechanism (M) that might result in different outcomes, including undersupply or the need for specific care strategies (O). For instance, in a given contextual environment, a certain UMR psychosocial context comprising asylum and youth care law alters care conditions and constraints, “which then triggers mechanism(s) that produce both intended and unintended outcomes. Intervention X may work well in one context but poorly or not at all in another context” [24]. An example is the undersupply that can occur when the treatment is not youth- or migration-sensitive.

The goal is to visualise CMO configurations [25], knowing that underlying mechanisms may be hidden [24, 26]. According to realist guidelines, [20, 27] this RR applies six stages: (1) identifying the review question, (2) formulating an initial theory, (3) searching and selecting primary studies, (4) extracting evidence, (5) appraising study quality, and (6) synthesising relevant and contradictory data [20, 27, 28]. Resulting in an evidence-based framework, an initial theory will be tested, substantiated and refined by empirical data. As “realist-informed” [21], this RR is consistent with the RAMESES publication standards (Supplementary file 1) for realist syntheses [24].

### Stage 1: identifying the review question

The question is *how* PSC works (C) and *why* specific mechanisms (M) determine (un-)intended PSC outcomes (O), conditioned by asylum and youth care policies.

### Stage 2: formulating an initial theory

**Fig. 1:**
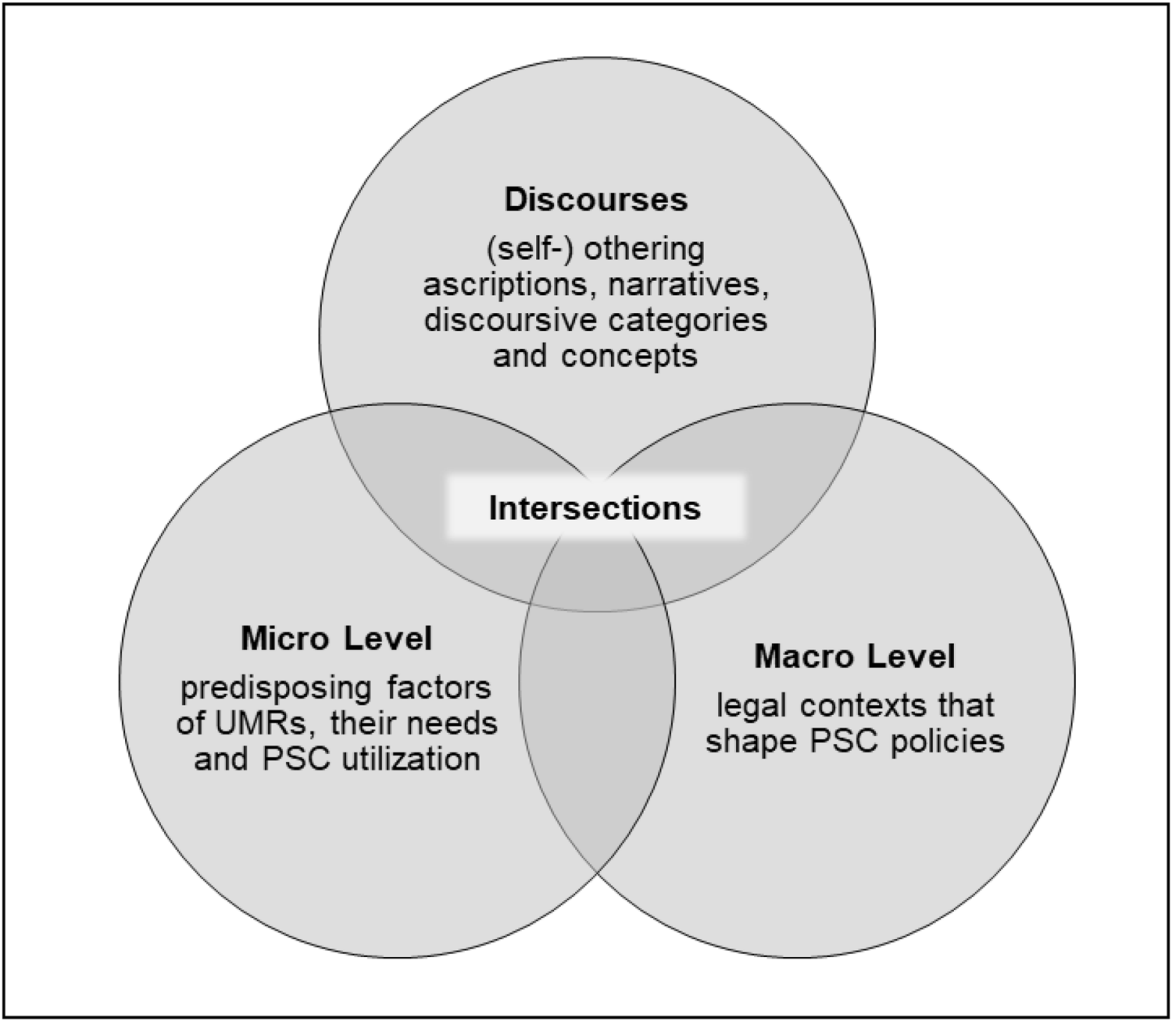
Initial theory on PSC for UMRs

The underlying initial theory model serves as the first orientation tool of the method. As a starting point in our attempt to outline the contexts and conditions of PSC, it functions to subsequently compare the findings of the literature review. The theory aims to produce a hypothesis with an innovative character. The theory model contains a three-dimensional conceptualisation based on micro-, macro- and discursive levels. Thus, these three levels include subjective characteristics of UMRs and social phenomena in everyday life at the micro-level and political regulations that determine their PSC system at the macro-level. Discourses and stereotypes—depending on how something is talked about or defined—which form social life, comprise an intermediate level in the model (shown in the pyramid as a peak). (Fig. 1).

The general division between micro- and macrostructural levels of PSC allows the inclusion of the UMRs’ protective and risk factors, social conditions, societal surrounding (such as living conditions) and psychological health. All these phenomena arise and have effects on the micro-level as well as on the macro-level.

These macro and micro factors can be mediated by daily and specialised UMR-related discourses and concepts. Such a theory must reflect the PSC needs of UMRs and the context factors, including assimilation and acculturation theories [29]. The theory building offers a comprehensive sociological,process-related model that seeks to refine the key premises, general conditions and modes of operation of PSC.

First, the micro-level of the initial theory consists of predisposing factors, PSC needs and utilisation. Taking into account the variety of different spatial and gender-specific contexts, utilisation as a microsocial factor must be included in the preliminary considerations of an initial theory [30].

The micro-level of the initial theory is moreover combined with theories of developmental psychology as well as a flight phase model that might include preflight experiences (e.g., war). Insights into the psychological processes of personal development and its challenges are given by Rossman et al. [31]. The theory model’s editing information concerning the phases of flights is provided by the flight phase model, which connects flight experiences with a processual understanding of trauma [32]. Both models can nurture the proceeding by giving insights into the UMRs’ specific challenges related to their young age as well as the particularities of their flight experience [33].

Second, within the theoretical framework, the individual level is fundamentally linked with macrostructural factors, such as health and migrations policies, on different regulatory levels.

Third, another element of the theory construct covers the discourses and concepts of PSC for UMRs and links micro- and macrosocial outcomes. Here, the theory aims at capturing intersections of individual, structural, and discursive contexts that influence PSC. This part is strongly inspired by approaches and recent debates of intersectionality and othering discourses that focus on public and migrant health [34–36]. The inclusion of intersectional perspectives enables the model to consider overlapping inclusions and exclusions, i.e., discrimination and privileges. The approach can also be helpful with regard to public and global health research that focuses on care situations for migrants and refugees [37].

Within the focussed discourses, othering denotes a sociopsychological differentiation process from people (or the self) in relation to the host community. In this process, migrants are perceived as “others” and as alienated from the in-group (e.g.,german host society).

For example, the sequential trauma concept (as part of special scientific discourses) that understands trauma as processual and contextual [38] everyday discursive events, such as othering and narratives about UMRs, are conceptually included. The barriers of PSC might then become effective on a general language-based, cultural or juridical level.

The first overview of the possible conditions and mechanisms of the PSC of UMRs now offers the starting point for the literature search. The theoretical considerations are first kept in mind and are presented again in the evaluation of the search results, which makes it possible to compare the preliminary considerations with the results. Therefore, the aim of the RR is to explain different findings from the literature on different theoretical bases and not to only use one initial theory as a basis for interpretation.

### Stage 3: Searching and selecting primary studies

To refine the initial theoretical framework, based on a search query for empirical evidence dating from 2005 until 2019, the studies’ search was performed between January and March 2019. The publication period was chosen because of the particular interest in the European migration context since 2005, the year when the council of the European Union published a directive on the minimum standards for the procedures for granting and withdrawing refugee statuses [39]. Since then, the directive has become effective as a set of common rules for dealing with asylum seekers across the EU.

Using the electronic databases Social Sciences Citation Index (Web of Science), PubMed/MEDLINE and GBV (German library network), the primary research articles and review studies were chosen and considered to be of the highest relevance to the scope and focus of the study. The RR focuses on the PSC situation of UMRs in Germany, not least due to Germany’s special position as a receiving immigration country in 2015 and 2016. Still, the body of literature, which includes important theoretical approaches from the pan-European context, and empirical data are included.

Search terms pertaining to UMRs in all topics and fields in articles in the above-mentioned electronic databases were chosen and included. First, common acronyms for UMRs and Unaccompanied Refugee Minors as well as *Unbegleitete Minderjährige Ausländer* were searched in the following style: “UMR” OR “URM” OR “UMA”. A second step included broadening the search by using fully written forms in a single search procedure: “asylum seeker OR asylum-seeking OR refugee OR migrant OR displaced OR Flüchtling OR Geflüchtet AND Minor OR young OR adolescent OR minderjährig OR junge OR youth AND Unaccompanied OR separated OR unbegleitet”.

**Fig. 2:**
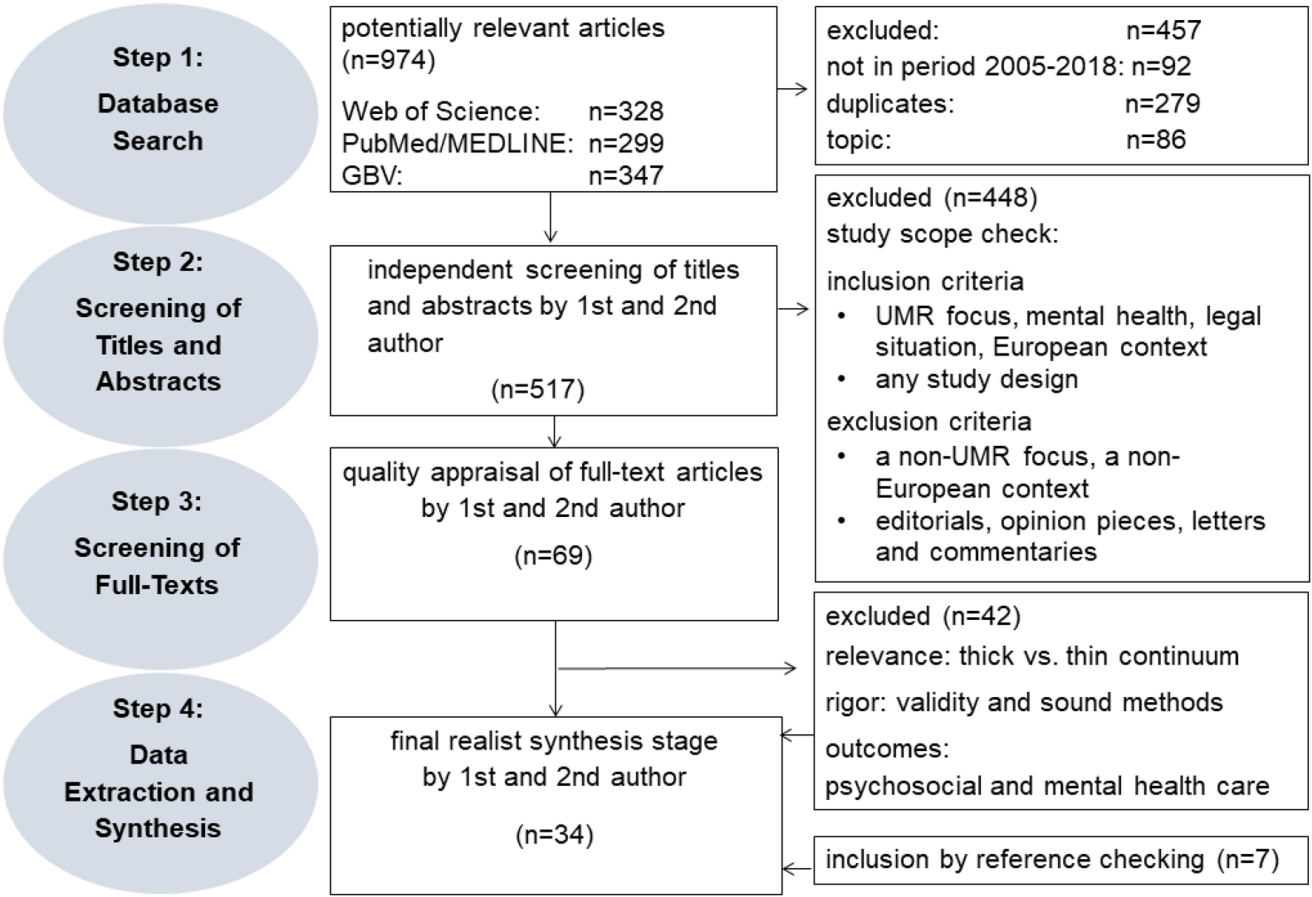
Flowchart Stage 5: appraising study quality

In total, 974 potentially relevant titles were imported into the electronic literature database (Fig. 2). The articles’ exclusion due to their allegiance to the research period and the deletion of duplicates led to 517 left publications. Non-English or non-German articles were excluded (*n* = 5) because of the focus explained above, and the large majority of the screened evidence was published in peer-reviewed English and German language journals.

With regard to their relevance for the study scope and their matching against the exclusion criteria displayed in Table 2, these 517 titles and abstracts were screened and independently checked by HSU and EK. Based on the previously mentioned criteria, 448 studies were excluded, as the main focus of these studies was not UMRs, their mental health (as for psychological problems and psychiatric symptoms), their legal situation or the European context. The non-European evidence was excluded. The excluded literature mainly focused on refugees in general, accompanied minor refugees or on refugees in the South American and Oceanic contexts. Moreover, editorials, letters and commentaries were excluded.

**Table 2:** Inclusion and exclusion criteria used for the screening of the study’s scope: inclusion/exclusion criteria

Study’s focus	Includes UMR Mental health (as for psychological problems and psychiatric symptoms) Legal situation European context
	Excludes UMR focus without a medical or psychosocial context Non-UMR focus (e.g., adult refugee or accompanied asylum seeker) Non-European context
Study design	Includes Any design (qualitative, quantitative, reviews) Non-peer-reviewed documents (gray literature)
	Excludes Editorials Mass media and social media Letters and commentaries
Outcomes	Includes Barriers in psychosocial care Sociological outcome: discriminatory narratives and discourses Medical outcomes: barriers in health services
	Excludes No information on sociological or medical policy outcomes

For full-text screening, the selection included 34 articles (Fig. 2); articles based on the “realist review’s focus on relevance and rigor.” were chosen. This required an appraisal of the “study quality” [24]. In a realistic synthesis, relevance assesses whether an article is capable of contributing to the construction of CMOs, while rigor influences the validity and trustworthiness of the evidence. The screened empirical evidence was tested, substantiated, and refined with CMO configurations regarding the PSC of UMRs. The results relate to outcomes pertaining to PSC barriers, sociological outcomes (e.g., discourses) and medical outcomes (Table 2), such as the levels of access and (health) care claims of UMRs. In total, 7 articles were added by reference checking, and 42 texts were dropped, as their relevance was too thin and their rigor was found not to be valid enough for the study’s scope. For example, some of the excluded studies were mainly concerned with medical issues, such as tuberculosis, or juridical details around asylum law.

### Stage 4: extracting evidence

Finally, 34 articles that contained empirical evidence of sufficient rigor and relevance were retained, and these articles were included in the extracting and synthesising process (Fig. 2). Studies on the psychological and mental well-being of UMRs were dominant (*n* = 21). A MAXQDA document served as a variables’ manager, by which for each article, eight categories were applied to document characteristics, such as study design, data or methods, journal or publisher, year, country, scientific discipline, relevance and rigor [21]. This document is provided in Supplementary file 2. To ensure collaborative and gender-sensitive insights, one male and two female sociologists, namely, HSU, EK and MM, interpreted the data. Furthermore, by collaboratively comparing the topics and theoretical approaches, the empirical evidence was checked against the theoretical frameworks, to potentially integrate it into a refined theory and final framework.

### Stage 5: appraising study quality

The final screening of full-texts functioned as a quality appraisal process for the evaluation of relevance, rigor and outcomes. Using MAXQDA software [40], HSU openly and axially coded the extracted evidence, and after the content was allocated to codes within categories, EK and MM carried out a subsequent and collaborative review of the generated code and category system. Followed by qualitative (*n* = 6), quantitative approaches (*n* = 4), mixed-methods designs (*n* = 3), and monographs (*n* = 3), reviews and systematic syntheses (*n* = 15) were used in the majority of the selected articles. The relevance of the selected papers was classified by the “thick/thin continuum” [21], which refers to a paper’s density of evidence regarding CMOs that are relevant to the study’s scope. *Thick* titles included rich and detailed accounts of relevant CMOs pertaining to PSC among UMRs, whereas *thin* contributions offered sparse data [21, 28]. *Thin* articles predominantly measured just one relevant aspect, such as psychological interventions for UMRs, without revealing much about mechanisms that could have mediated any observed effect and could have provided a wider context. By taking into account the rigor and richness of the evidence, the relevance of the selected papers was categorised as a thick, moderate or thin contribution [21, 28]. The multiple occurrences of the term “UMR” as well as the potential of the texts to provide extensive or specific knowledge about the sought-after topic’s rigor and richness were measured constantly. Qualitative and mixed-methods studies tended to provide thicker evidence than did quantitative works, whose content was predominantly moderate or thin with respect to relevant CMO compositions (Supplementary file 2).

### Stage 6: synthesising relevant and contradictory data with propositions

Four steps were taken to synthesise the final empirical evidence. First, based on the extraction of the interrelated CMOs—concepts explaining mechanisms-, the superordinated CMO propositions were formulated. As a kind of summary heading, these propositions did not entirely reflect all mechanisms, but they were “close enough to observed data to be incorporated in propositions that permit empirical testing” [41].

Second, according to a realist synthesis, HSU identified mechanism patterns, also known as “demi-regularities” [28], which are “semi-predictable patterns or pathways of programme functioning” [25]. What is meant here are findings from processes described in the literature, which then constitute a starting point for an assumption that other comparable processes could proceed in a similar way. These observed patterns were formulated into continuously revised and refined realist CMOs, such as “If programme activity (PA) xn + 1 in context (C) xn + 1, then mechanism (M) xn + 1 is put in place that will then lead to outcome (O) xn + 1 [20].” These patterns are represented in process formulations in CMO form.

Third, the formulated sub-processes and mechanisms summarizing CMO propositions were again checked by re-reading the papers and considering the initial theory. Throughout the synthesis process, they were tested with primary evidence and against contradictory findings [21, 28]. Finally, HSU, EK and MM checked whether the evidence was used properly in the interpretative synthesis, and after discussions, all authors agreed upon potential misreadings and ambiguities [28, 42].

## Results

**Fig. 3:**
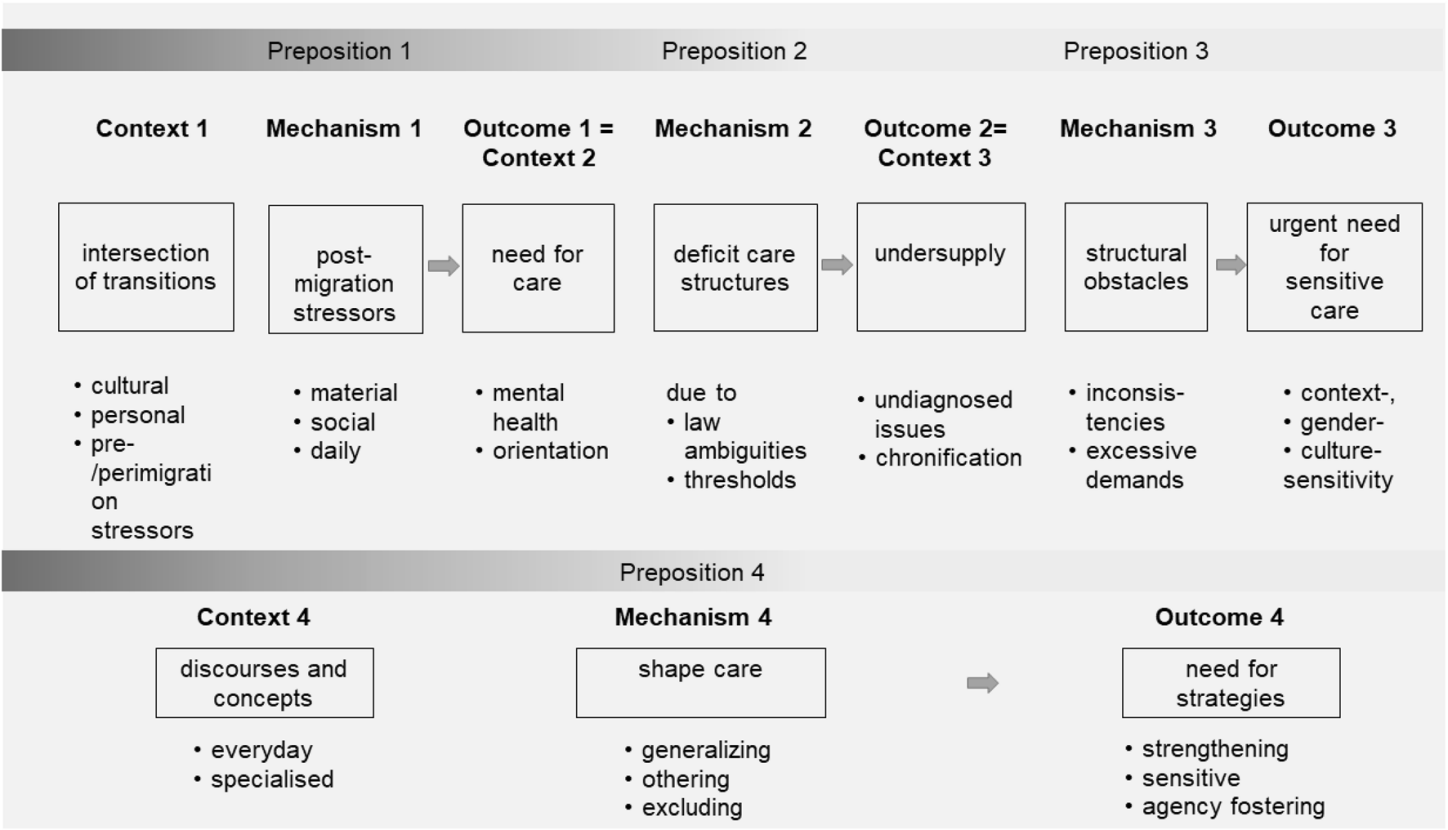
For UMRs, the final PSC framework including contexts, mechanisms and outcomes

Characterised by their interrelatedness, the dominant, multiple contexts, mechanisms, and outcomes (CMO propositions) turned out to be the following:

- the intersections of transitions (e.g., of adolescence and migration);
- the need for context-, culture-, and gender-sensitive mental and PSC; and
- the undersupply within PSC (see Fig. 3).

These contexts and outcomes are mediated by pre-, peri- and post-migratory stressors as well as care structures and are, moreover, influenced by overarching discourses and concepts (CMO proposition 4 in Fig. 3). They comprise adverse and beneficial mechanisms in the PSC of UMRs.

### Proposition 1

If (C1) the processes of migration and adolescence define an intersection of transitions for UMRs, post-migratory stressors could be triggered (M1) and may lead to a need for psychosocial care (O1).

The UMRs’ situation can be interpreted as an in-between state of continuous cultural adjustment due to their migration experience [43] and of contemporaneous personal development due to their adolescent age [33, 44]. The flight itself might cause a faster simultaneous development of autonomy [33] and dependencies, while orientations between autonomy and bonding have to be balanced. Overcoming language barriers in the country of arrival is one of the cultural adjustments that increases stress factors [45, 46]. Moreover, premigration experiences [47, 48], a perimigration burden [49] and traumatic experiences, such as continuing existential insecurities, can be part of their layered processes of change. The general developmental tasks can be complicated by the superimpositions of acculturation and flight processes [33].

This intersection of transitions functions as a risk factor for PTSD [49] and at the same time demands multiple adjustment and coping strategies [46, 50]. Post-migratory stressors are multifaceted [51] and shape the UMRs’ daily lives, psychosocial well-being and mental health; they include material, social and daily stressors as well as acculturational stress. Especially, constant negotiations with authorities and a general lack of residence stability can lead to the UMRs’ lack of trust in the arriving societies and especially in its care systems [46, 52].

Because trust is of high importance and functions in a life-guiding and even safety-ensuring role in the UMRs’ pre- and peri- migration phases [46, 53, 54], a lack of trust might impact their agency and future orientations [52]; this may aggravate their adjustment processes and shape their PSC, psychological and psychiatric therapy needs [55]. Access to trustworthy care structures is interpreted as giving a “sense of belonging” [56]. The stigmatisation of mental health care needs as well as the cultural specificity of health concepts and understandings of illness [48, 54] further define the mechanisms that shape care needs. When these needs are not recognised, the risks for late-onset illness, especially PTSD [45, 57], might occur and lead to further needs for psychiatric care, psychosocial orientation and stability. As the above reviewed literature argues, a refugee youth-specialised, trust-building and agency fostering PSC might work best for UMRs.

### Proposition 2

If UMRs have a psychosocial care need (C2) and are confronted with deficient care structures (M2), an undersupply of the UMRs psychosocial health care (O2) could occur.

Social and political conditions of arrival are part of living conditions and post-migratory stressors that contribute to the already vulnerable and partially traumatised state of some UMRs [53, 58]. The arrival phase marks an important period in the processing of the experiences of settling in the new environment [51]. On the one hand, social and cultural adjustment is highly linked to the personal as well as the social background and motivation of the UMRs themselves and their attitude towards their new living conditions [46]; on the other hand, it is also linked to the existence of either welcoming or othering cultures within the arrival country [6]. The political contexts concerning the right to family reunification and the classifications of UMR countries of origin as safe or unsafe define further mechanisms [49, 52] that shape care contexts, as described above. Possibly undersupply and failures to recognise the UMRs’ needs [46] and barriers to PSC can be reinforced by the general ambiguity between human and children´s rights and asylum law [49]. For example, within the German context, the conflict between the youth welfare legislation (SGBIII) and the German Asylum Laws (AsylG) put caregivers in disempowering situations, which consequently promote risks for PTSD chronification and the undersupply of PSC for UMRs [49, 52, 59]. Due to inappropriate legal conditions, there is a certain risk of administrative detention throughout Europe and especially Germany [60]. These conflicts relate to unclear responsibilities within youth welfare, questions concerning family reunions [61] and protection needs of UMRs [62, 63, 63]. Especially regarding the importance of the arrival phase as a time in which UMRs need to find stability and in the meaningful phase of processing traumatic events, their adjustment can be hindered or impeded by law-related ambiguities [51]. During this period, the need for psychotherapeutic or psychiatric interventions can be intensified. Low-threshold offers in these fields cannot alleviate the underlying structural conditions but can provide partial relief. Another outcome is gender and relates to gender-specific reasons to flee [44] (e.g., FGM female genital mutilation [49, 52]). One of the multiple reasons the female proportion of UMRs is relatively low in the arriving countries is the fact that girls face multiple dangers of sexual exploitation during their migration process and beyond (in Germany, of the total UMRs, girls comprised 8,6% in 2015 [52] and 10% in 2016 [44]). Because of gender-specific flight experiences and care needs in the arrival countries, girls have a vulnerability that forms a context. Due to male-specific or gender non-sensitive PSC strategies, female UMRs´needs are barely addressed and with that gender forms an outcome at the same time. Gender specificity also applies to acculturation processes and social roles, as women experience shifting gender norms and roles during and after their migration [64]: numerous different facets of transition can thereby be demonstrated in the case of female UMRs. The question of gender-specific care programs and their use by female UMRs could not be explained by the literature reviewed.

### Proposition 3

The existing undersupply of psychosocial health care for UMRs (C3) and further adverse structural obstacles (M3) might lead to an urgent need for sensitive care strategies and concepts (O3).

Concerning the PSC structures and policies, again, the German context offers an example of the European experience of UMR care-specific realities. In 2015, the institutional and social structures in the field of youth welfare services, administration, schools and psychosocial centres faced the requirement for immediate establishment that was appropriate for new structures and for the consolidation of existing ones. Amongst others, these excessive demands were mediated and handled by individuals, such as counsellors of psychosocial centres and became part of interdisciplinary and provisional care networks [65].

Due to inconsistencies of structural conditions [58] (across regions and cities, administrative units with different population densities), there was a need for a mapping of the existing care structure across places and regions. Deprived regions and rural areas, in particular, had to overcome excessive demands resulting from shifting UMRs to institutions of established care structures within metropolitan areas (such as Berlin). This transfer resulted in excessive demands for well-established existing structures and an with that an undersupply of PSC for UMRs on the ground. Psychiatric institutions for children and young people, in particular, had to compensate for this undersupply situation [66]. In addition, high thresholds to care form barriers that might lead to missing diagnoses or possible chronifications [67, 68]. Those structural barriers include residency permits, persisting asylum procedures, unclear or inconsistent care practices, missing cost coverages, language barriers and the absence of language mediation and stigmatisation of mental health issues [69–71]. New care facilities are, therefore, needed and should comprise sustainable care, legal frameworks, the language and cultural skills of caregivers, clear and sufficient financing, time resources for personnel, and less administrative efforts for care processes and interventions [58].

### Proposition 4

Everyday, lay and specialised expert discourses and concepts (C4) might have a (non-)protective effect on UMRs (O4) and should be approached in a context-sensitive manner (M4).

Discursive labelling and the contextual classification of UMRs are sociologically, empirically, legally and morally rooted [49] and render a connection of migration, migration policies, national legislation, international conventions and human rights standards with ideas about UMRs´ needs, legitimacy and hopes. Declaring the possible family reunion as a danger [61] and therefore strengthening othering processes [49], socially rooted narratives such as the concept of so-called anchor children (as children that are sent by their parents to improve the family´s economic status) contribute to the degradation of the social recognition of UMRs. Everyday discourses and narratives, such as the idea of so-called anchor children, may function as othering mediators [46] and frame UMRs with negative stereotypes [49, 51]. This may cause the UMRs psychosocial adjustment to be impeded, their own expectations for adjustment to be challenged and may even lead to self-othering [46]. Negative stereotypes and stigmatisation of mental disorders and psychiatric care can also reduce the mental health of UMRs and their use of services. Low-threshold PSC services can be the first step into mental health care and pave the way to recovery.

As more male UMRs migrate and international conventions are not gender-specific, although the interplay of asylum status and gender causes multiple protection needs for female UMRs [44, 63]. By classifying the countries of origin as (un-)safe, the legal foundations such as migration policies define the legitimacy of the UMRs’ claims of asylum [49]. On the other hand, morally rooted preconceptions of children as individuals in need of protection lead to considering UMRs as individuals in need of this protection.

The overarching everyday and scientific discourses thereby in manifold ways shape the UMRs’ PSC and their social positions. A frequently phrased and used framework, namely, the dichotomy of vulnerability and resilience, is common within psychological- and mental health-related publications on UMRs [6, 72] as well as the expert jargon. The ambivalence of these approaches and interpretations of UMRs becomes relevant when the consequences of their application are considered. A generalisation of the UMRs’ resilience might lead to an overestimation of their capacities to process traumatic experiences. Short-term interventions, thereby possibly further on provoke chronification and unmet care needs. On the other hand, a generalisation of vulnerability might lead to an underestimation of the UMRs resources, strengths or capacities [73] and may function as a barrier in the context of successful resource-oriented interventions [61]. UMRs might identify with their own vulnerability and have difficulties adjusting in the arriving countries.

As the proposition suggests, specialised expert discourses and concepts have effects on UMRs and care-specific practices. Professional approaches to UMRs and related psychological trauma concepts might counter those effects and operate inclusively [49]. A resource-oriented understanding of posttraumatic stress disorders can foster meaning and comprehension of trauma-related behavior, such as intrinsic mechanisms or dissociative situations, and help the UMRs’ agency development, resilience and adjustment [68]. The concept of sequential traumatisation positions trauma on a social, political and time dimension, which allows opening up psychological frames to events of structural discrimination in host societies as well as seeing trauma as multiple events in a life-phase process [74]. However, the concept and psychological application of the sequential trauma model [33, 75] is not considered in the majority of the UMR-related psychological literature [3, 67, 76]. If care and care strategies are informed by these concepts, the UMRs’ agency can be promoted. Working with young UMRs could be turned into a trauma-sensitive activity if trauma is recognised and understood as a process that can continue to the present state. Furthermore, keeping their “as well as” state between resilience and vulnerability in mind, a need-orientated and individualised care might support the UMRs and represents a type of care in which UMRs are not generalised as a homogeneous group [76]. As age, gender and the number of traumatic events of UMRs represent PTSD risk factors [51], the application of an intersectional approach considers forms of discrimination in every phase of migration and widens the perspectives of mental health understandings. Higher acceptance and trust towards the care system can then, in turn, improve the PSC for UMRs.

## Discussion

This is the first realist-informed synthesis of the UMRs most recent PSC situations in high-income countries in the European Union, specifically Germany. The findings reveal that the UMRs’ life stage is deeply characterised by intersecting transitions of migration and adolescence and confronted with personal and societal obstacles that might be enforced by binary and generalising discourses around them.

PSC is shaped by policy regulations located between international human rights and domestic asylum laws and the direct microsocial outcomes of that interaction (e.g., an undersupply that can deepen chronification). In addition, the general discursive level is particularly significant, as the everyday concepts and expert jargon shape PSC and its processes. This review has shown that deficient PSC contexts, underlying structures and mechanisms can lead to fundamental gender-specific and socio-spatial care barriers ( e.g., in metropolitan areas such as Berlin): If those barriers are not balanced out by care strategies and networks at the local level, further PSC needs might be consolidated. The following paragraph first summarises the RR’s findings and provides an interpretation of the results of the CMO constellations in their propositions. Furthermore, the strengths and weaknesses of the RR approach in relation to the chosen topic are discussed. To be able to establish a link to PSC in practice, recommendations for action and implications conclude the discussion.

### Key findings and interpretation

This study investigated the *mechanisms* (M) of PSC programs within different *contexts* (C) and the programs’ multiple *outcomes* (O).

Proposition 1 explained the connection between the intersecting processes of migration and adolescence and post-migratory stressors as well as the resulting PSC needs of UMRs. Their health care needs are examined by various disciplines, such as health service research. Therefore this review argues to link theoretical approaches such as the flight phase model with approaches from developmental psychology [33]. The special need for psychosocial care as previously described in proposition 1 has already been suggested by Huemer, who compared UMRs and accompanied minor refugees. Her study showed the importance of social support (e.g., family) for young migrating people and provided indications on how social contact and family support act as protective factors [47].

Proposition 2 and 3 propose that an undersupply of health care and further needs for sensitivity are possible outcomes from the cumulation of existing PSC needs and deficient care structures. Psychosocial undersupply is highly complex and might have several impacts upon UMRs, such as a missed diagnosis of PTSD, inadequate housing conditions or potential social isolation [29]. As demonstrated in this RR, multiple factors such as trust, culture, language and gender specificities meet UMRs within different phases of migration [49, 54].

Proposition 4 discussed discourses and concepts, their effect on UMRs and their care. The synthesis of several studies [2, 49, 51] showed that the concept of sequential trauma may serve as a comprehensive and suitable tool for capturing the UMRs’ mental health and their PSC contexts. Furthermore, generalising concepts such as “vulnerability” or “resilience” [1] should be critically reflected within everyday practices and academic research. Amidst increasing numbers of arriving UMRs to Germany and other European countries, a critical reflection of concepts and discourses is crucial [6]. In summary, UMRs are said to be in age- and migration-specific transition processes; thus, special PSC needs arise. A possible shortage of PSC has various consequences for the well-being of UMRs. Within the care system, it is not only a question of the structural existence of care facilities but also of sensitive care concepts.

### Strengths and limitations

In this RR scientific studies, gray literature and monographs dealing with PSC were extensively analysed with the aim to identify CMOs. Given the common perception that gray literature is “not evidence-based”, it is less likely to be used as source for articles published in peer-reviewed journals. Yet, its inclusion enhanced our understanding of hidden mechanisms. Another strength comprises the given overview of the processes and PSC outcomes. It suggests the inclusion of intersectional approaches as innovative tools to unveil discriminatory structures and practices [35, 36, 77]. It indicates that sociological approaches can enrich epidemiological research as well as monocausal approaches of migrant health research.

The limitations of this work are related to the high level of abstraction [20], which is applied in the preparation of the initial theory and in the refined framework. Consequently, second- and third-order interpretations (such as the development of CMOs and its propositions) can merely have a reduced claim for translation into PSC practice. Due to the broad variety of reviewed literature across disciplines and research topics as well as different prevalence data the empirical evidence of the CMOs is inconclusive. For instance, the available data on PTSD indicate a prevalence range between 19 and 54% [51]. Another limitation includes the focus on literature written in English and German; this restriction is due to our own language barriers (e.g., lack of Arab, French, Turkish, or Russian literacy). The fact that the evidence is regionally and nationally specific and that there is a high variability and diversity of the PSC programs can also be listed as a limitation within the given RR. Nevertheless, the RRs methodological approach and the given results allow the development of PSC practical recommendations that can be applied across European countries and especially in the German context.

### Recommendations and implications

The following recommendations regarding UMRs and their PSC focus on practical care strategies and the academic field of health service research [26, 78]. On the practical level, PSC might be informed by a resource-oriented, individualised care approach based on trust. When UMR agencies are strengthened, the UMRs’ self-efficacy and autonomy can be fostered [33]. Care strategies that recognise the UMRs’ structural dependency on bureaucratic procedures and attempt to seriously understand the complexity of social dependencies (e.g., local care networks, educational institutions such as schools) might be effective. There is a reciprocal relation of psychosocial and thus psychiatric care institutions. Psychosocial services and counselling can function as gatekeepers to psychiatric care and lead to low-threshold services. In contrast, psychiatric services and care can be complemented by psychosocial services, and a release from short-term intensive psychiatric care to other care structures can be supported.

Research strategies on UMRs should be shaped and informed by intersectional approaches [79] that provide ways of interpreting the multiple discriminations, privileges and complexities of social integration. Approached as being embedded in diverse social categories (as minor, (un-)accompanied, (non-)religiously or culturally diverse) UMRs might receive a more differentiated contextualisation by health service research and social sciences.

Last, research can further enrich the practical aspects of PSC, if caregivers, institutional actors and the scientific sphere see the importance of a practice-theory exchange. Therefore, scientific projects and practical care could integrate intersectionality with regards to potentially discriminatory practices in migrant health service [80–82], such as age assesments in the arrival phase.

Finally, the political circumstances shaping UMR policies and PSC regulations are based on asylum status´ and legitimacies of access to PSC. When these policies and legal structures contribute to the existing traumatisation and PTSD among UMRs, they promote othering and victimisation [35]. Humane, health equity-oriented and inclusive policies can meet the special needs of UMRs by perceiving their particular situation of dependency and at the same time by promoting their resources through shaping integrative and empowering psychosocial and social settings. Moreover, the intersections of inclusion and exclusion call for non-discriminatory treatment, universal access opportunities beyond citizenship, and individualised provision of support.

## Conclusions

The review has shown that the existing body of literature grasps the PSC of UMRs by different disciplinary angles and approaches but does not offer a comprehensive overview on the micro–macro intersections and included discourses. The inclusion of lay perspectives and an intersectional approach could inform health service research and practitioners. The reflection of the UMR’s social positions between categorical constructs of resilience and vulnerability, the discriminatory discourses of othering, and the restrictive health policies may guide policy recommendations to potentially reduce the persistent disadvantages and PSC barriers as well as improve PSC practices. This might help to develop appropriate UMR-targeted PSC that promotes agency strengthening as well as autonomy fostering.

## Supplementary Information

Below is the link to the electronic supplementary material.

Supplementary file1 (PDF 668 KB)

## Data Availability

Not applicable.
