# Supplementary material for: Explaining psychosocial care among unaccompanied minor refugees: a realist review
Source: Eur Child Adolesc Psychiatry. 2021 Mar 29;31(12):1857–70. doi: 10.1007/s00787-021-01762-1 (PMC9663343; doi:10.1007/s00787-021-01762-1)
Supplement: Supplementary file 1 — Supplementary file1 (PDF 668 KB) [file 787_2021_1762_MOESM1_ESM.pdf]

Supplementary File 1: RAMESES Guidelines for realist syntheses – checklist:
Wong G, Greenhalgh T, Westhorp G, et al. RAMESES publication standards: realist syntheses. BMC Med 2013;11:21, pp. 4.

Section/topic	#	Checklist item	Reported on page #
**TITLE**			
Title	1	In the title, identify the document as a realist synthesis or review.	**cover page (1)**
**ABSTRACT**			
	2	While acknowledging publication requirements and house style, abstracts should ideally contain brief details of: the study's background, review question or objectives; search strategy; methods of selection, appraisal, analysis and synthesis of sources; main results; and implications for practice.	**2**
**INTRODUCTION**			
Rationale for review	3	Explain why the review is needed and what it is likely to contribute to existing understanding of the topic area.	**2-3**
Objectives and focus of review	4	State the objective(s) of the review and/or the review question(s). Define and provide a rationale for the focus of the review.	**3**
**METHODS**			
Changes in the review process	5	Any changes made to the review process that was initially planned should be briefly described and justified.	**3**
Rationale for using realist synthesis	6	Explain why realist synthesis was considered the most appropriate method to use	**3-4**
Scoping the literature	7	Describe and justify the initial process of exploratory scoping of the literature	**4-7**
Searching processes	8	While considering specific requirements of the journal or other publication outlet, state and provide a rationale for how the iterative searching was done. Provide details on all the sources accessed for information in the review. Where searching in electronic databases has taken place, the details should include, for example, name of database, search terms, dates of coverage and date last searched. If individuals familiar with the relevant literature and/or topic area were contacted, indicate how they were identified and selected.	7-10
Selection and appraisal of documents	9	Explain how judgements were made about including and excluding data from documents, and justify these.	**11-12**
Data extraction	10	Describe and explain which data or information were extracted from the included documents and justify this selection.	**13**

Supplementary File 1: RAMESES Guidelines for realist syntheses – checklist:
Wong G, Greenhalgh T, Westhorp G, et al. RAMESES publication standards: realist syntheses. BMC Med 2013;11:21, pp. 4.

Analysis and synthesis processes	11	Describe the analysis and synthesis processes in detail. This section should include information on the constructs analyzed and describe the analytic process.	**13-14**
**RESULTS**			
Document flow diagram	12	Provide details on the number of documents assessed for eligibility and included in the review with reasons for exclusion at each stage as well as an indication of their source of origin (for example, from searching databases, reference lists and so on). You may consider using the example templates (which are likely to need modification to suit the data) that are provided.	**12 (Figure 2)**
Document characteristics	13	Provide information on the characteristics of the documents included in the review.	**10 (Table 1)**
Main findings	14	Present the key findings with a specific focus on theory building and testing.	**14-20**
**DISCUSSION**			
Summary of findings	15	Summarize the main findings, taking into account the review's objective(s), research question(s), focus and intended audience(s).	**21-23**
Strengths, limitations and future research directions	16	Discuss both the strengths of the review and its limitations. These should include (but need not be restricted to) (a) consideration of all the steps in the review process and (b) comment on the overall strength of evidence supporting the explanatory insights which emerged. The limitations identified may point to areas where further work is needed.	**23-24**
Comparison with existing literature	17	Where applicable, compare and contrast the review's findings with the existing literature (for example, other reviews) on the same topic.	**22-24**
Conclusion and recommendations	18	List the main implications of the findings and place these in the context of other relevant literature. If appropriate, offer recommendations for policy and practice.	**24-26**
**FUNDING**			
Funding	19	Provide details of funding source (if any) for the review, the role played by the funder (if any) and any conflicts of interests of the reviewers.	**cover page (1)**

Author	Design	Data/Methods	Title	Journal/Publisher	Year	Country	Scientific discipline	Relevance	Rigor
Abbas et al.	Review		Migrant and refugee populations: a public health and policy perspective on continuing global crisis	Antimicrobial Resistance & Infection Control	2018	Switzerland	Epidemiology	medium	high
Agbih et al.	Review		Gesundheitsversorgung für Flüchtlinge aus ethischer Perspektive: Wo fangen die Fragen an?	Die kosmopolitische Klinik: Globalisierung und kultursensible Medizin	2017	Germany	Philosophy	medium	medium
Bean et al.	Quanitative Study Paper	n=582; Follow up after 12 months, focusing well-being, need and utilization of mental health services	Course and predictors of mental health of unaccompanied refugee minors in the Netherlands	Social Science and Medicine	2007	Netherlands	Mental Health	thick	high
Bean et al.	Quanitative Study Paper	n=920 UMRs, n=557 legal guardians, n= 496 teachers; focusing on well-being, need and utilization of mental health care services in comparison with Dutch sample (n = 1059)	Factors associated with mental health service need and utilization among unaccompanied refugee adolescents	Administration and Policy in Mental Health and Mental Health Services Research	2006	Netherlands	Mental Health	thick	high
Bröse et al.	Monograph		Flucht. Herausforderungen für soziale Arbeit	Springer VS	2017	Germany	Social Work	thick	medium
Bünnemann et al.	Review	n=5; Interviews with experts and guardians	Entwicklung und Aufbau eines niedrigschwelligen psychologischen Versorgungsangebots für	Zeitschrift für Klinische Psychologie und Psychotherapie	2018	Germany	Mental Health	thick	high

			unbegleitete minderjährige Flüchtlinge						
Curtis et al.	Review		Migrant children within Europe: a systematic review of children's perspectives on their health experiences	Public Health	2018	UK	Health Science	medium	mediu m
Derluyn et al.	Review		Psychosocial wellbeing of `Vulnerable` Refugee Groups in (Post-) Conflict Contexts: An Intriguing Juxtaposition of Vulnerability and Resilience	Springer International	2019	Belgien	Mental Health	medium	high
Efler	Monograph		Unbegleitete minderjährige Flüchtlinge. Kinder- und Jugendliche im Spannungsfeld zwischen dem SGB VII und dem deutschen Ausländerrecht	GRIN-Verlag	2014	Germany	Law	low	high
Eide, Hjern	Report		Unaccompanied refugee children- vulnerability and agency	Acta Pædiatrica	2013	Sweden	Mental Health	thick	high
Groark et al.	Qualitative Study Paper	Interpretative Phenomenologica l Analysis	Understanding the experiences and emotional needs of unaccompanied asylum-seeking adolescents in the UK	Clinical Child Psychology and Psychiatry	2011	UK	Mental health	thin	high
Habib et al.	Review		Gender role changes and their impact on Syrian women refugees in Berlin in light of the Syrian crisis	WZB Berlin Social Science Center	2018	Germany	Sociology/Gender Studies	thin	high
Hargasser	Review		Unbegleitete minderjährige Flüchtlinge. Sequentielle	Brandes& Apsel	2016	Germany	Mental Health	thick	high

			Traumatisierungsprozesse und die Aufgaben der Jugendhilfe						
Hodes et al.	Review		Challenges and opportunities in refugee mental health: Clinical, service, and research considerations	European Child & Adolescent Psychiatry	2018	Germany	Mental Health	medium	medium
Huemer	Report		Unaccompanied Refugee Children	Lancet	2009	Germany	Mental Health	thick	high
Keilson	Quanitative Study Paper		Sequentielle Traumatisierung bei Kindern: Untersuchungen zum Schicksal jüdischer Kriegswaisen.	Edition psychosozial	2005	Netherlands	Mental Health	thin	high
Majumder et al.	Qualitative Study Paper	n=15; Exploration of personal views on mental health	This doctor, I not trust him, I'm not safe: the perceptions of mental health and services by unaccompanied refugee adolescents	International Journal of Social Psychiatry	2014	UK	Mental Health	high	high
Metzner et al.	Review		Psychotherapeutic treatment of accompanied and unaccompanied minor refugees and asylum seekers with trauma-related disorders in Germany	Bundesgesundheitsblatt-Gesundheitsforschung-Gesundheitsschutz	2016	Germany	Mental Health	thick	high
Namer et al.	Review		Settling Ulysses. An Adapted Research Agenda for Refugee Mental Health	Health Policy Management	2017	Germany	Mental Health	medium	high
Nesterko et al.	Qualitative Study Paper	Two commented Case Reports	Kultursensible Aspekte während der Diagnostik von psychischen Belastungen bei Flüchtlingen – Zwei kommentierte	Zeitschrift für Klinische Psychologie und Psychotherapie	2017	Germany	Mental Health	medium	high

			Fallberichte						
Nosè et al.	Report		Access to mental health services and psychotropic drug use in refugees and asylum seekers hosted in high-income countries	Epidemiology and Psychiatric Sciences	2015	Sweden	Mental Health	high	high
Nowotny et al.	Review		Medizinische Versorgung von Kindern und Jugendlichen mit Fluchthintergrund	Jugendmedizin	2018	Germany	Health Science	low	high
Papadopoulos	Report		A Psychosocial Framework for with Refugees	self-published report/gray literature	2011	UK	Mental Health	medium	high
Piesker et al.	Qualitative Study Paper	Concept of sequential traumatization connected to psychological wellbeeing of UMRs in three case reports	Sequentielle Traumatisierungsprozesse bei unbegleiteten Minderjährigen Geflüchteten	Trauma und Gewalt	2018	Germany	Mental Health	thick	high
Rania et al.	Qualitative Study Paper	n= 10; Perceptions of migration process of UMRs, semi-structured interviews	Unaccompanied Migrant Adolescents in the Italian Context: Tailored Educational Interventions and Acculturation Stress	Child and Youth Services	2018	Italy	Social Work	low	high
Rücker et al.	Mixed Methods	n=52; Refugee Health Screening	Resilient or Risk Group? Psychological Burden at Unaccompanied Refugee Minors (URM) in Germany	Praxis der Kinderpsychologie und Kinderpsychiatrie	2017	Germany	Mental Health	thick	high
Sanchez-Cao et al.	Quanitative Study Paper	N=71; Harvard Trauma Questionnaire, Strengths and Difficulties Questionnaire,	Psychological distress and mental health service contact of unaccompanied asylum-seeking children	Child: care, health and development	2013	UK	Mental Health	medium	high

		Impact of Event Scale, Birleson Depression Self-Rating Scale for Children, Attitudes to Health and Services Questionnaire							
Sierau et al.	Report		Herausforderungen im Fluchtprozess unbegleiteter Jugendlicher	Kindheit und Entwicklung	2019	Germany	Mental Health	high	high
Spallek et al.	Mixed Methods	Two Qualitative Interviews, one Quantitative Study	Gesundheitliche Situation und Versorgung unbegleiteter minderjähriger Flüchtlinge- eine Näherung anhand qualitativer und quantitativer Forschungen in der Stadt Bielefeld	Bundesgesundheitsblat-Gesundheitsforschung-Gesundheitsschutz	2016	Germany	Epidemiology	medium	high
Thomas et al.	Monograph		Unbegleitete minderjhrige Geflüchtete. Ihre Lebenssituationen und Perspektiven in Deutschland	transcript Verlag	2018	Germany	Sociology	thick	high
Vervliet et al.	Quanitative Study Paper	n=103; Follow-up after 18 months, focusing on mental health and daily stressors	The mental health of unaccompanied refugee minors	Eur Child Adolesc Psychiatry	2014	Belgium	Mental Health	thick	high
Vervliet et al.	Mixed Methods	Self-report Questionnaire	Multilayered Ethics in Research Involving Unaccompanied Refugee Minors	Journal of Refugee Studies	2015	Belgium	Social Science	low	mediu m
Vervliet et al.	Quanitative Study Paper	Self-report Questionnaire,	Longitudinal follow-up of the mental health of	European Child & Adolescent Psychiatry	2014	Belgium	Mental Health	high	mediu m

		follow-up	unaccompanied refugee minors						
Wernesjö	Review		Unaccompanied asylum-seeking children: Whose perspective?	Childhood	2012	Sweden	Mental Health	medium	high

Supplementary File 2: MAXQDA variables manager document/ document listing the MAXQDA variables of all reviewed articles
